# Neoantigen reactive T cells correlate with the low mutational burden in hematological malignancies

**DOI:** 10.1038/s41375-022-01705-y

**Published:** 2022-10-08

**Authors:** Sunil Kumar Saini, Staffan Holmberg-Thydén, Anne-Mette Bjerregaard, Ashwin Unnikrishnan, Simon Dorfmüller, Uwe Platzbecker, Irene Tirado-Gonzalez, Halvard Bönig, Daniel El Fassi, Kirsten Grønbæk, John Pimanda, Hind Medyouf, Sine Reker Hadrup

**Affiliations:** 1 Department of Health Technology, Section of Experimental and Translational Immunology, Technical University of Denmark, Kongens Lyngby, Denmark; 2 Adult Cancer Program, Lowy Cancer Research Centre, UNSW, Sydney, NSW 2052 Australia; 3 Prince of Wales Clinical School, UNSW, Sydney, NSW 2052 Australia; 4 Medical Clinic and Policlinic 1, Hematology and Cellular Therapy, Leipzig University Hospital, Leipzig, Germany; 5 Institute for Tumor Biology and Experimental Therapy, Georg-Speyer-Haus, Frankfurt, Germany; 6 German Red Cross Blood Service and Institute for Transfusion Medicine and Immunohematology of the Goethe University, Frankfurt, Germany; 7 Department of Clinical Medicine, University of Copenhagen, Copenhagen, Denmark; 8 Department of Hematology, Herlev and Gentofte Hospital, Herlev, Denmark; 9 Department of Hematology, Copenhagen University Hospital, Copenhagen, Denmark; 10 Department of Hematology, Rigshospitalet, Copenhagen, Denmark; 11 Biotech Research and Innovation Centre, Faculty of Health and Medical Sciences, University of Copenhagen, Copenhagen, Denmark; 12 The Danish Stem Cell Center (Danstem), Faculty of Health and Medical Sciences, University of Copenhagen, Copenhagen, Denmark; 13 Department of Clinical Medicine, Faculty of Health and Medical Sciences, University of Copenhagen, Copenhagen, Denmark; 14 Hematology Department, South Eastern Area Laboratory Services, Prince of Wales Hospital, Randwick, NSW Australia

**Keywords:** Immunosurveillance, Immunosurveillance, Antigen presentation, Myelodysplastic syndrome, Cytotoxic T cells

## To the Editor:

Myelodysplastic syndrome (MDS) is a disease characterized by cytopenia, bone marrow dysplasia, and clonal hematopoiesis resulting from acquired mutations in hematopoietic stem cells, with a median of nine somatic mutations per exome, or ~1500 in the entire genome [1]. The mutational burden is related to its disease severity, with a lower number of mutations in low-risk MDS and higher numbers in high-risk disease and secondary acute myeloid leukemia [1, 2]. It is, however, substantially lower than the number of mutations found in most other types of cancer [3].

There is strong evidence that cancer types with higher mutational burden respond better to T lymphocyte (T cell) dependent therapies such as adoptive cell transfer and checkpoint inhibition [3, 4]. The mechanism behind this finding, derives from the capacity of mutations to give rise to neoantigens. Neoantigens can lead to T cell-mediated tumor cell killing since mutated DNA is translated to peptides that are foreign to the immune system, and hence, recognized by T cells when presented on human leukocyte antigen (HLA) molecules. It has been questioned whether the low mutational burden in MDS is sufficient to generate neoantigens to trigger cancer-specific T cell responses. Researchers have demonstrated that only a minor fraction of tumor mutations are recognized by T cells, and a higher mutational burden may consequently increase the chance of such T cell recognition to occur [5]. Current approved treatments for MDS are limited to allogeneic bone marrow transplantation, DNA methyltransferase inhibitors (DNMTi), lenalidomide, or supportive care. Advances to improve survival for this patient group are highly needed.

## Detection of neoantigen-specific T cells in MDS

To evaluate the immune system’s ability to recognize mutation-derived neoantigens that are likely to presented on the cell surface of malignant cells in MDS patients, we used a combined bioinformatics and laboratory screening approach to identify specific T cell populations that bind neoantigen-HLA complexes. Using whole-exome DNA and mRNA sequencing data from CD34+ bone marrow cells and fibroblasts, we identified mutation-derived peptides and predicted their HLA-binding capacity to HLA class I in each individual [6]. Peptides were then ranked based on their peptide-HLA-binding affinity.

**Fig. 1:**
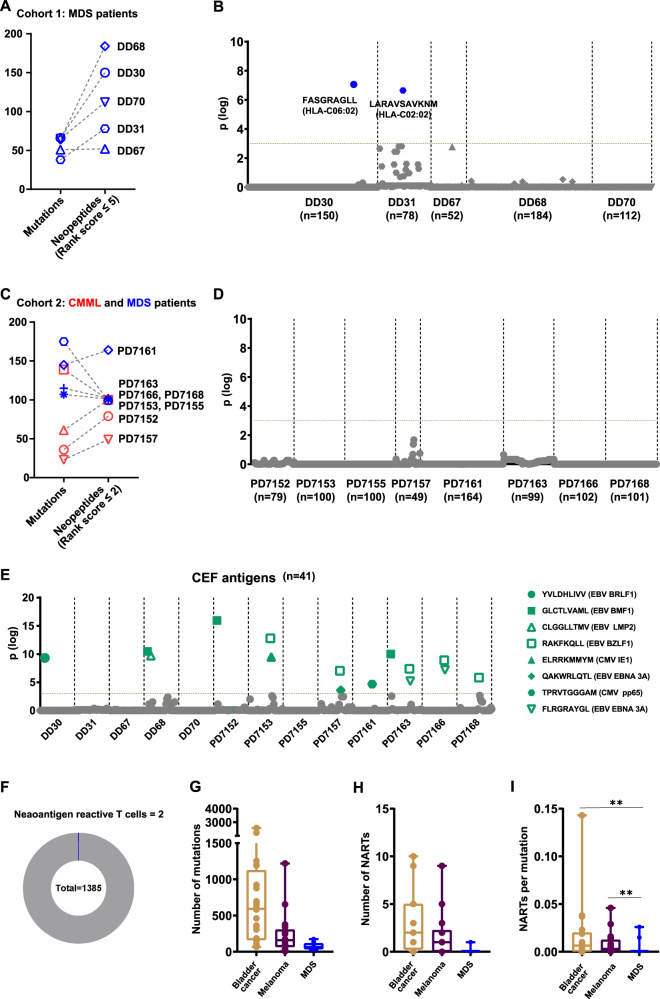
Neoantigen reactive CD8 T cells identified in hematological malignancies. **A** Plot showing the number of mutations and their corresponding predicted neoantigens for patients in the first MDS cohort. *n* = 5; mutations (mean = 57, SD = 11), neoantigens (mean = 115, SD = 53). **B** Neoantigen binding CD8 T cells identified using DNA-barcoded MHC multimers in individual patient samples from the first cohort. CD8 T cell recognition to individual neoantigens was identified based on the enrichment of DNA barcodes associated with each of the tested peptide specificities (LogFc > 2 and *p* < 0.001, barracoda). Significant T cell recognition (above the horizontal dotted line) of individual peptide sequences is shown in blue color (neoantigen-specific). Patient ID and the number of neoantigens analyzed are labeled on the *X*-axis. Vertical dotted line separate data for the individual patient samples. Non-significant responses are shown in gray. **C** Plot showing the number of predicted neoantigens selected for experimental evaluation in the second patient cohort. Cohort 2 (*n* = 8); netMHCpan 4.0 peptide rank score ≤2 or minimum of 100 peptides. Mutations (mean = 100, SD = 55), neoantigens (mean = 100, SD = ±32). Blue symbols represent MDS patients, while red symbols are CMML patients. **D** Similar to (**B**), Neoantigen binding CD8 T cells identified in the second cohort. **E** *CEF*-reactive T cells identified in the two MDS cohorts. Significant responses are shown in green, and epitope specificities are described in the legends. **F** Pie chart showing the fraction of neoantigen reactive CD8 T cells among the total analyzed neoantigens. **G** Boxplot with number of mutations (single nucleotide variants, deletions, and frameshift) in MDS (*n* = 15, mean ± SEM; 77 ± 12) compared with bladder cancer (*n* = 24, 738 ± 142) and melanoma (*n* = 43, 668 ± 109). **H** Number of neoantigen reactive T cells identified in melanoma and bladder cancer cohorts using the same method used for the MDS patients. **I** Neoantigen reactive T cells identified in bladder cancer, melanoma, and MDS patients, normalized to the total number of mutations found in the respective patient. Mann–Whitney test; *p* = 0.002 (MDS and Melanoma), *p* = 0.002 (MDS and Bladder).

In a cohort of five patients with high-risk MDS (Fig. 1A, Supplementary table 1), with an average of 57 (SD = 11) mutations in their coding regions, a total of 576 potential HLA-matching neoantigens were identified with a peptide-binding score of ≤5 (rank score, netMHCpan) [7]. In a second cohort of eight patients (Supplementary table 2; four high-risk MDS and four chronic myelomonocytic leukemia type II, CMML-II) with a higher mutational burden (mean 100 mutations, SD = 55), 783 neoantigens were identified with a rank score of ≤2 or a maximum of 100 peptides per patient (Fig. 1C, Supplementary table 3). To identify CD8 T cells reactive to the selected neoantigens, bone marrow aspirates from the individual patients were analyzed using DNA-barcoded MHC-I (major histocompatibility complex class I) multimers (Supplementary fig. 1) [8]. Patient-specific neoantigen peptides were loaded to matching MHC-I molecules and multimerized on a fluorophore-labeled dextran backbone tagged with a unique DNA barcode. T cell populations binding to the peptide-MHC-multimers were then isolated using fluorescence-activated cell sorting, and their specificity was revealed by amplification and sequencing of the co-attached DNA barcodes. Sequencing data were analyzed using the software package Barracoda (version 1.8) (http://www.cbs.dtu.dk/services/Barracoda). As an experimental control, MHC multimers loaded with 41 known viral epitopes of cytomegalovirus, Epstein-Barr virus, and influenza (Flu) virus (CEF, Supplementary table 4) were also included in the analysis.

On average, we analyzed 101 neoantigens per patient (range = 52–182). We identified CD8 + T cells reactive to the neoantigens only in two of the 13 analyzed patients (Fig. 1A–D). CEF-reactive T cells were identified in nine patient samples validating the analysis platform (Fig. 1E). Of the 1359 neoantigens analyzed across the 13 patients, only two were recognized by the CD8 T cells (Fig. 1F), one each in patients DD30 and DD31. Both the immunogenic neoantigens were restricted to HLA-C molecules; FASGRAGLL to HLA-C06:02 (DD30) and LARAVSAVKNM to HLA-C02:02 (DD31). The two immunogenic peptides were derived from a frameshift mutation in RUNX1 and a missense mutation in AP3S1 respectively (Supplementary Table 5). Compared to patients with melanoma and bladder cancer, where neoepitope-specific T cell recognition had similarly been evaluated [9, 10], it is observed that the MDS cases have significantly fewer mutations and predicted neopeptides (Fig. 1G–H). This furthermore resulted in fewer neoepitope-reactive T cell populations, even when normalized for the difference in tumor mutational burden (Fig. 1I). T cell responses were only detected in two of our 13 MDS patients, a striking difference compared to melanoma and bladder cancer patients, where T cell reactivity was observed in 31 of 42 patients evaluated using the same strategy. The data suggest that the generation of an immunogenic neoepitope is a relatively rare event and that a substantial mutational burden is likely required to obtain neoantigen-mediated tumor cell recognition.

**Fig. 2:**
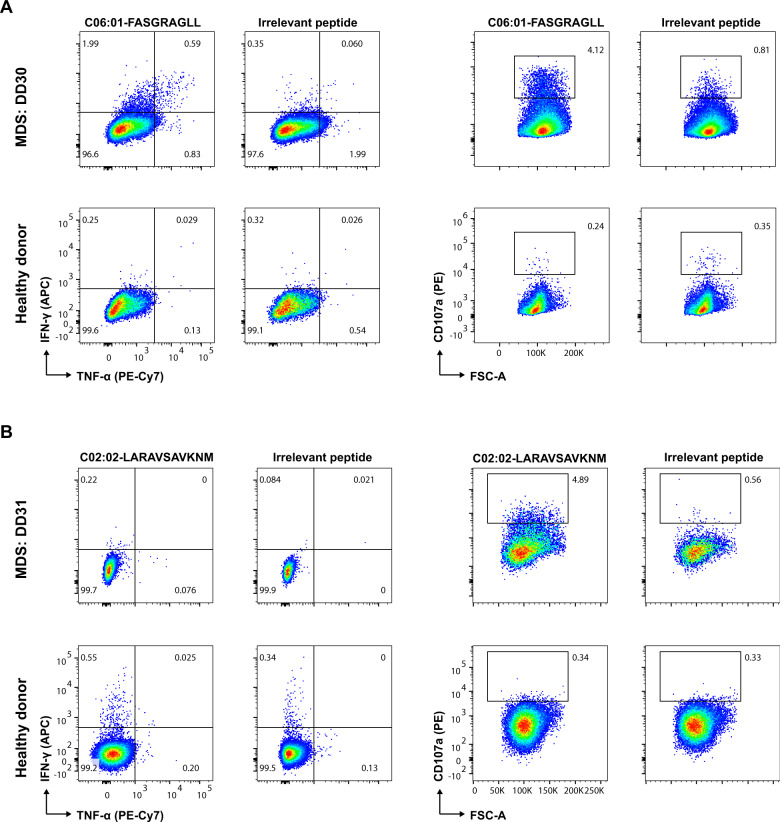
Functional evaluation of T cell reactivity toward neoantigens. **A** Flow cytometry dot plots showing intracellular cytokine staining measuring Interferon-gamma (IFN-γ) and Tumor necrosis factor-alpha (TNFα, left), and CD107a (right) in the CD8 + T cells stimulated with the neoantigen FASGRAGLL, identified to recognize CD8 T cells in MDS patient DD30 (Fig. 1D), or an irrelevant peptide (HLA-C06:01 restricted). Comparative cytokine release profile is shown for cells expanded from the MDS patient DD30 (upper row) and a healthy donor (bottom row). **B** Similar to (**A**), intracellular cytokines data measured after stimulation with HLA-C02:02 restricted neoantigen LARAVSAVKNM (identified to recognize CD8 T cells in MDS patient DD31, Fig. 1D). The numbers on the plot indicate the % frequency of CD8^+^ T cells double or single positive for the analyzed cytokines.

Next, we validated the presence of neoepitope-specific T cells by functional recognition of the two neoepitopes. First, the bone marrow-derived T cells were pre-incubated with peptide-HLA complexes and cytokines to expand antigen-specific T cells to relevant levels for functional evaluation. The cells were then stimulated with the neoepitopes to evaluate cytokine secretion upon antigen recognition (see supplementary method for details). In patient DD30, increased interferon-γ secretion and increased CD107a expression, a marker for degranulation, was observed when T cells were stimulated with FASGRAGLL compared to an irrelevant peptide (Fig. 2A). In patient DD31, T cell stimulation with LARAVSAVKNM increased CD107a expression compared to stimulation with an irrelevant peptide (Fig. 2B). Peptide stimulation before the expansion of neoantigen-specific T cell populations showed an increased interferon-γ signal in T cells but unchanged CD107a expression compared to the cells stimulated with an irrelevant peptide. No cytokine staining experiment was performed pre-expansion in DD30 due to poor viability of the bone marrow cells in this patient. Comparative experiments using peripheral blood mononuclear cells from a healthy donor, cultured with the same peptide-HLA complexes as DD30 and DD31, did not show an increased interferon-γ response or increased expression of CD107a when stimulated with the peptides described above (Fig. 2).

## Discussion

We here report the ex-vivo detection of neoepitope T cell recognition in the bone marrow of MDS patients. Our study is among the first to report that functional neoantigen-specific T cells can be detected in MDS patients, demonstrating that despite the low-mutational burden in MDS, T cell recognition of mutation-derived antigens can occur. The two neoantigens recognizing T cells identified in this study resulted both from a well-characterized driver gene in myeloid neoplasms (RUNX1; patient DD30) and from a mutation in AP3S1 gene (patient DD31) not known for its role in pathogenesis, which suggests that neoantigens can arise from both driven and passenger mutations in this malignancy.

The level of recognition observed here, i.e., neoepitope-reactive T cells detected in two out of 13 patients, is lower than similar evaluations in high mutational burden cancer. A recent study in melanoma and bladder cancer demonstrated neoepitope T cell recognition in 13 out of 18 melanoma patients and 18 of 24 patients with bladder cancer before treatment with immunotherapy [9, 10]. Our data represents a relatively small patient cohort, despite this limitation, the findings presented here support the earlier notion that the chance of finding a neoantigen-reactive T cell population correlates to the mutational burden of the tumor (Fig. 1G–I) [11]. The low frequency of specific T cells found in this study could have implications for the development of immunotherapy in MDS patients. For example, therapies intended to stimulate T cells to target malignant hematopoietic stem cells might not be able to rely on neoepitopes to drive the immune response, which has been recognized as the primary mechanism of action for checkpoint inhibition in diseases with a high mutational burden [4].

Likewise, a recent study by Ferrari et al. evaluated the neoepitope T cell reactivity in five MDS patients [12]. 21 somatic variants were identified that could induce 31 distinct T cell populations when blood samples were stimulated with peptide-pulsed dendritic cells in vitro. Of the 31 neoantigen-specific T cell populations detected, 22 were also able to lyse autologous tumor cells in a tumor cell killing assay. The successful induction of neoantigen-specific T cells led to initiation of a phase I clinical trial, where three patients with MDS were treated with expanded autologous neoantigen-specific T cells [13]. This indicates that low levels of neoantigen reactive T cells are present in MDS, at frequencies undetectable with current technology, and that these T cell populations can be enhanced by using immunotherapies that specifically boost such T cell responses.

Even though neoantigen reactive T cells are sparse in MDS patients, there are reasons to believe that checkpoint inhibitors still could have a role to play in the disease. Combining checkpoint inhibition with epigenetic therapy, such as DNMTi, could facilitate T cell reactivity against antigens that are upregulated by the epigenetic therapy, such as cancer-testis antigens or elements from endogenous retroviruses [14, 15]. Similarly, antigens derived from aberrant expression of certain proteins such as cancer-testis antigens (WI1, NY-ESO-1, PRAME1, etc.) could also serve as therapeutic targets in the absence of neoantigens.

## Supplementary information

Supplementary information

Supplementary Table 3

## Data Availability

The data that support the finding of this study, in addition to the supplementary data, can be accessed from the corresponding author upon request.
