# Supplementary material for: Neoantigen reactive T cells correlate with the low mutational burden in hematological malignancies
Source: Leukemia. 2022 Oct 8;36(11):2734–8. doi: 10.1038/s41375-022-01705-y (PMC9613475; doi:10.1038/s41375-022-01705-y)
Supplement: Supplementary file 1 — Supplementary information [file 41375_2022_1705_MOESM1_ESM.docx]

**Supplementary information**

**Neoantigen reactive T cells correlate with the low mutational burden in hematological malignancies**

**Authors**:
Sunil Kumar Saini^1^*, Staffan Holmberg-Thydén^1^*, Anne-Mette Bjerregaard^1^, Ashwin Unnikrishnan^2,3^, Simon Dorfmüller^1^, Uwe Platzbecker^4^, Irene Tirado-Gonzalez^5^, Halvard Bönig^6^, Daniel El Fassi^7-9^, Kirsten Grønbæk^10-13^, John Pimanda^2,3,14^, Hind Medyouf^5^, Sine Reker Hadrup^1†^

^1^Department of Health Technology, Section of Experimental and Translational Immunology, Technical University of Denmark, Kongens Lyngby, Denmark.

^2^Adult Cancer Program, Lowy Cancer Research Centre, UNSW, Sydney, NSW 2052, Australia.

^3^Prince of Wales Clinical School, UNSW, Sydney, NSW 2052, Australia.

^4^Medical Clinic and Policlinic 1, Hematology and Cellular Therapy, Leipzig University Hospital, Leipzig, Germany.

^5^Institute for Tumor Biology and Experimental Therapy, Georg-Speyer-Haus, Frankfurt, Germany.

^6^German Red Cross Blood Service and Institute for Transfusion Medicine and Immunohematology of the Goethe University, Frankfurt, Germany.

^7^Department of Clinical Medicine, University of Copenhagen, Copenhagen, Denmark.

^8^Department of Hematology, Herlev and Gentofte Hospital, Herlev, Denmark.

^9^Department of Hematology, Copenhagen University Hospital, Copenhagen, Denmark.

^10^Department of Hematology, Rigshospitalet, Copenhagen, Denmark

^11^Biotech Research and Innovation Centre, Faculty of Health and Medical Sciences, University of Copenhagen, Denmark

^12^The Danish Stem Cell Center (Danstem), Faculty of Health and Medical Sciences, University of Copenhagen, Denmark

^13^Department of Clinical Medicine, Faculty of Health and Medical Sciences, University of Copenhagen, Denmark.

^14^Hematology Department, South Eastern Area Laboratory Services, Prince of Wales Hospital, Randwick, NSW, Australia

*These authors contributed equally to this work

†Corresponding author. Email: sirha@dtu.dk

# Supplementary table 1 - Patient characteristics, cohort 1

| **Patient** | **Disease (WHO Classification)** | **Gender** | **Age** |  | | | | **IPSS-R  ^(MDS)^ CPSS ^(CMML)^** | **BM Blast %** | **Mutations (total)** | **Missense** | **Insertion** | **Deletion** | **Frameshift** |
| --- | --- | --- | --- | --- | --- | --- | --- | --- | --- | --- | --- | --- | --- | --- |
|  |  |  |  | **Hb**  **(g/L)** | **WBC**  **(x10^9/L)** | **Pt (x10^9/L)** | **Cytogenetics** |  |  |  |  |  |  |  |
| DD30 | MDS RCMD | M | 56 | 136.8 | 3 | 49 | trisomy 8 | 6.5 Very High | 7% | 66 | 53 | 2 | 2 | 9 |
| DD31 | MDS RAEB-1 | F | 76 | 126.00 | 4.5 | 121 | NORMAL | 5.5 High | 11% | 38 | 32 | 0 | 2 | 4 |
| DD67 | MDS RAEB-2 | M | 51 | 131.4 | 8.80 | 15 | NORMAL | 6.5 Very High | 50% | 51 | 47 | 0 | 0 | 4 |
| DD68 | MDS RAEB | F | 78 | 117 | 8.73 | 163 | NORMAL | 3.5 Intermediate | 3,5% | 64 | 45 | 0 | 11 | 8 |
| DD70 | MDS RAEB-2 | F | 77 | 88.2 | 1.96 | 62 | NORMAL | low | 36% | 65 | 52 | 0 | 6 | 7 |

# Supplementary table 2 - Patient characteristics, cohort 2

| **Patient** | **Disease (WHO Classification ^^^)** | **Gender** | **Age** |  | | | | **IPSS-R ^$ (MDS)^ or CPSS^$$ (CMML)^** | **BM Blast %** | **Mutations (total)** | **Missense** | **Insertion** | **Deletion** | **Frameshift** |
| --- | --- | --- | --- | --- | --- | --- | --- | --- | --- | --- | --- | --- | --- | --- |
|  |  |  |  | **Hb**  **(g/L)** | **WBC**  **(x10^9/L)** | **Pt (x10^9/L)** | **Cytogenetics** |  |  |  |  |  |  |  |
| PD7152 | CMML | F | 65 | 91 | 54 | 442 | NORMAL | Score=2/Intermediate-2 | 4% | 36 | 33 | 0 | 0 | 3 |
| PD7153 | CMML | F | 70 | 90 | 4,1 | 20 | NORMAL | Score=1/Intermediate-1 | 2% | 139 | 131 | 3 | 4 | 1 |
| PD7155 | CMML | F | 70 | 87 | 26,46 | 15 | der(7) t(7;10) trisomy 8 | Score= 4/High Risk | 17% | 61 | 60 | 0 | 1 | 0 |
| PD7157 | CMML | M | 59 | 98 | 6,85 | 169 | NORMAL | Score=2/Intermediate-2 | 7% | 23 | 15 | 0 | 8 | 0 |
| PD7161 | MDS/RAEB-2 | F | 75 | 82 | 1,3 | 88 | NORMAL | Score=5/High | 10% | 145 | 134 | 0 | 3 | 8 |
| PD7163 | MDS/RAEB-2 | F | 69 | 88 | 3,4 | 32 | 11 q deletion. Loss of MLL signal (by FISH) | Score=5.5/High | 12% | 175 | 167 | 2 | 0 | 6 |
| PD7166 | MDS/RAEB-2 | M | 69 | 128 | 6,36 | 193 | trisomy 8 | Score=5/High | 13% | 115 | 83 | 0 | 3 | 29 |
| PD7168 | MDS/RAEB-2 | F | 77 | 84 | 3,8 | 115 | 46,XX,del(7)(q22),der(12)inv(12)(q13q24.1)t(5;12)(q12;q24.1)[8]/47,sl,+8[4]/46,sl,del(13)(q12q14)[3]/46,XX[8] | Score=8/Very High | 19% | 107 | 87 | 1 | 19 | 0 |

*AUSTRALIAN INDICATION*

- *Intermediate-2 and High-risk Myelodysplastic Syndromes (MDS) according to the International Prognostic Scoring System (IPSS)*
- *Chronic Myelomonocytic Leukemia [CMML (10%-29% marrow blasts without Myeloproliferative Disorder)]*

*REFERENCES*

***^****Vardiman JW, Harris NL and Brunning RD. The World Health Organization (WHO) classification of the myeloid neoplasms. Blood 100, 2292-2302 (2002)*

***$****Greenberg, P. L. et al. Revised international prognostic scoring system for myelodysplastic syndromes. Blood 120, 2454-2465 (2012)*

***#****Cheson, B. D. et al. Clinical application and proposal for modification of the International Working Group (IWG) response criteria in myelodysplasia. Blood 108, 419-425(2006)*

***$$****Such et al. Development and validation of a prognostic scoring system for patients with chronic myelomonocytic leukemia. Blood 121, 3005-3015 (2013)*

# Supplementary table 4 – List of CEF peptides

| **S. No.** | **HLA type** | **Peptide** | **Peptide sequence** |
| --- | --- | --- | --- |
| 1 | HLA-A*01:01 | CMV pp65 YSE | YSEHPTFTSQY |
| 2 | HLA-A*01:01 | CMV pp50 VTE | VTEHDTLLY |
| 3 | HLA-A*01:01 | FLU BP-VSD | VSDGGPNLY |
| 4 | HLA-A*02:01 | FLU MP 58-66 GIL | GILGFVFTL |
| 5 | HLA-A*02:01 | EBV LMP2 CLG | CLGGLLTMV |
| 6 | HLA-A*02:01 | EBV BMF1 GLC | GLCTLVAML |
| 7 | HLA-A*02:01 | EBV LMP2 FLY | FLYALALLL |
| 8 | HLA-A*02:01 | CMV pp65 NLV | NLVPMVATV |
| 9 | HLA-A*02:01 | EBV BRLF1 YVL | YVLDHLIVV |
| 10 | HLA-A*02:01 | CMV IE1 VLE | VLEETSVML |
| 11 | HLA-A*02:01 | HIV Pol (C20) | ILKEPVHGV |
| 12 | HLA-B*07:02 | CMV pp65 TPR | TPRVTGGGAM |
| 13 | HLA-B*07:02 | CMV pp65 RPH-L | RPHERNGFTV |
| 14 | HLA-B*07:02 | EBV EBNA RPP | RPPIFIRLL |
| 15 | HLA-B*08:01 | Flu NP (C8) | ELRSRYWAI |
| 16 | HLA-B*08:01 | EBV BZLF1 (C9) | RAKFKQLL |
| 17 | HLA-B*08:01 | CMV IE1 | ELRRKMMYM |
| 18 | HLA-B*08:01 | EBV EBNA 3A (C10) | QAKWRLQTL |
| 19 | HLA-B*08:01 | EBV EBNA 3A (C11) | FLRGRAYGL |

# Supplementary table 5 – List of known pathogenic mutations

| **Cohort** | **Patient ID** | **Mutations** | **Comments** |
| --- | --- | --- | --- |
| Cohort 1 | DD30 | ZRSR2 p.Q234* | Oncogenic variant |
|  |  | EZH2 p.C329Y | Oncogenic variant |
|  |  | TET2 p.Q278* | Oncogenic variant |
|  |  | CEBPA p.L324P | Likely pathogenic |
|  |  | ASXL1 p. G646W | Oncogenic Variant |
|  |  | RUNX1 p. 356A | Oncogenic variant |
|  | DD31 | U2AF1 p.Q157P | Oncogenic Variant |
|  |  | ASXL1 p. G646W | Oncogenic Variant |
|  | DD67 | ASXL1 p. G646W | Oncogenic Variant |
|  | DD68 |  | no pathogenic mut |
|  | DD70 |  | no pathogenic mut |
| Cohort 2 | PD7152 | IDH2_p.R140Q | Oncogenic Variant |
|  |  | U2AF1_p.Q157R | Oncogenic Variant |
|  |  | JAK2_p.V617F | Oncogenic Variant |
|  | PD7153 | TET2_p.L1065fs*1 | Oncogenic Variant |
|  |  | TET2_p.Q685* | Oncogenic Variant |
|  |  | CUX1_p.Q1265* | Oncogenic Variant |
|  |  | SFRS2_p.P95H | Oncogenic Variant |
|  | PD7155 | DNMT3A_p.R882H | Oncogenic Variant |
|  |  | IDH2_p.R140Q | Oncogenic Variant |
|  |  | SFRS2_p.P95H | Oncogenic Variant |
|  | PD7158 | NF1_p.R1362* | Oncogenic Variant |
|  |  | ASXL1_p.E635fs*15 | Oncogenic Variant |
|  |  | NRAS_p.G13C | Oncogenic Variant |
|  |  | IDH1_p.R132C | Oncogenic Variant |
|  | PD7161 | TET2_p.K1491* | Oncogenic Variant |
|  |  | U2AF1_p.S34F | Oncogenic Variant |
|  | PD7163 | TP53_p.H179Y | Oncogenic Variant |
|  | PD7166 | SFRS2_p.P95T | Oncogenic Variant |
|  |  | CUX1_p.I946fs*46 | Oncogenic Variant |
|  |  | EZH2_p.K574E | Oncogenic Variant |
|  | PD7168 |  | no pathogenic mut |


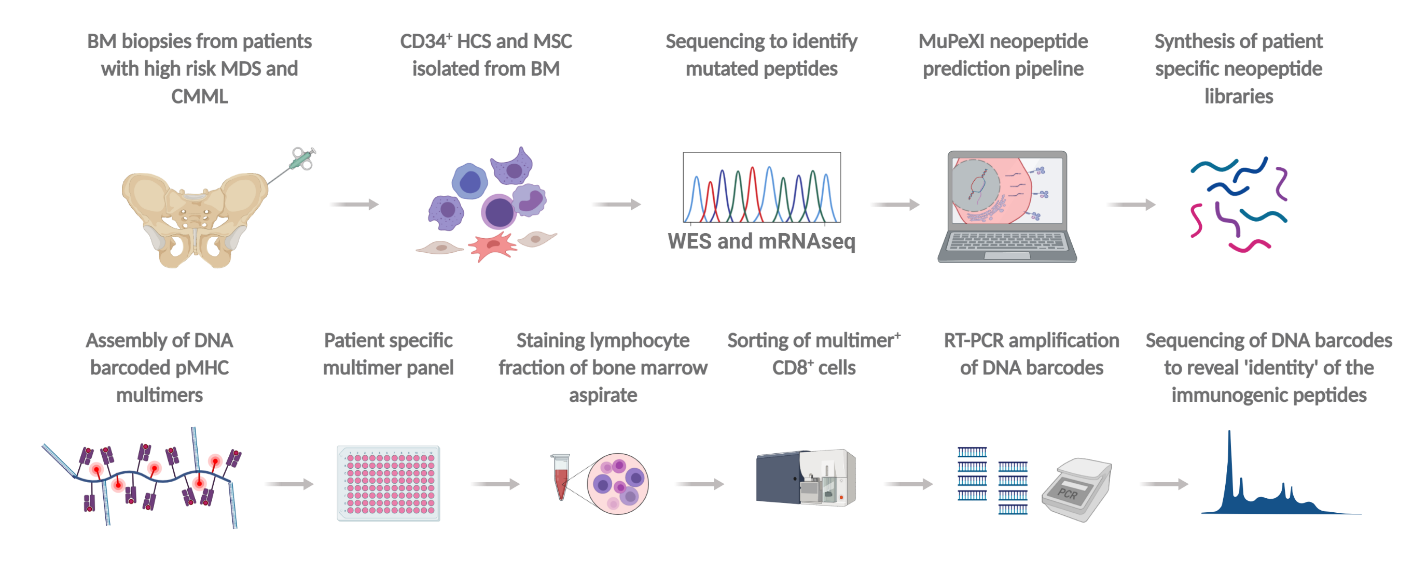


**Supplementary figure 1.** **Graphical representation of neopeptide selection and experimental evaluation to identify neoantigen-reactive CD8 T cells.** High throughput detection of neoantigen-specific T cells. CD34+ hematopoietic stem cells (HSC) and mesenchymal stem cells (MSC) were isolated from bone marrow aspirates (BM) of patients with MDS and CMML. HSC and MSC were sequenced using whole exome sequencing (WES) to identify somatic mutations of the malignant clones, while sequencing of mRNA was performed on the HCS compartment. Sequencing data was fed through a bioinformatics pipeline (MuPeXi and NetMHCpan) to identify which mutations were likely to be expressed and bound to HLA class I, on the malignant cells. Peptides with a low NetMHCpan rank score (≤ 5 using netMHCpan 2.8 for cohort 1, and ≤ 2 using netMHCpan 4.0 or maximum of 100 peptides) were synthesized to create libraries of patient-specific neopeptides. The neopeptides were loaded on matching HLA-monomers (pMHC), which then were multimerized on a dextran molecule with an attached DNA barcode and a fluorescent PE label. The panel of patient-specific pMHC-multimers was used to stain the BM of the corresponding patient, and multimer-positive CD8 cells were sorted into Eppendorf tubes. The barcodes attached to the sorted multimer-positive CD8 cells were amplified using RT-PCR and then sequenced to reveal the identity of the peptide responsible for the T cell binding. Figure created using biorender.com

# Supplementary methods

## Patients and bone marrow samples

Bone marrow samples included in this study were from two cohorts. The first one consisted of five patients with high-risk MDS, that were treated at the Department of Hematology and Oncology at the University Hospital in Mannheim, Germany. The second cohort included MDS patients enrolled in a clinical trial in Sydney, Australia. All samples were approved by regional ethics committees and had been collected following informed written consent, in accordance with the Helsinki declaration. Peripheral blood mononuclear cells from anonymous healthy donors were included as control.

## Peptides and neoantigen prediction

Whole exome sequencing (WES) and mRNA sequencing data from either CD34+ bone marrow cells, or whole bone marrow, together with WES from fibroblasts, were fed into *Mutant peptide extractor and informer* (MuPeXI) software, in order to predict which mutations that give rise to immunogenic peptides attached to HLA class I on the malignant cells. The peptides with the strongest predicted binding capacity to their respective HLA, based on the lowest rank score from NetMHCpan (version 2.8 for cohort 1, and version 4.0 for cohort 2) were synthesized by Pepscan Presto BV (Lelystad, The Netherlands). All peptides went through quality control analysis with UV and mass spectrometry, prior to release.

## Detecting specific T cells with barcode labelled multimers

### Generating peptide-MHC multimers

Heavy chains and beta-2-microglobulin for MHC class I molecules were produced in Escherichia coli and harvested using inclusion bodies. During the folding process, an exchangeable UV-sensitive peptide ligand was introduced to the MHC binding groove. Biotinylated unique DNA barcodes were created by combining different A and B oligos, so that each peptide investigated in the study could be represented by a specific barcode tag. Each specific barcode was then attached to a dextran molecule containing multiple streptavidin binding sites and a PE fluorescent label (Fina BioSolutions, Rockville, MD, USA). HLA monomers, corresponding to the patient’s tissue type, were loaded with the peptides of interest by UV-mediated exchange, which breaks down the ligand in the HLA peptide groove when exposed to UV radiation, and replaces it with a peptide with high affinity for the specific HLA-type. The peptide-MHC complexes were then attached to barcode-labeled dextrans, through biotin-streptavidin conjugation.

### Identification of antigen-specific T cells

Bone marrow samples were thawed and incubated with a pooled library of the barcoded peptide-MHC multimers, to allow for interaction between antigen-specific T cells and peptide-MHC complexes. Cells were then stained with a mix of fluorescent antibodies containing CD8-PerCP (Invitrogen), viability dye (LIVE/DEAD Fixable Near-IR; Invitrogen), and a FITC dump channel: CD4-FITC (BD), CD40-FITC (Serotech), CD19-FITC (BD), CD16-FITC (BD), and CD14-FITC (BD). Cells stained with surface markers and peptide-MHC multimers were fixed in 1% paraformaldehyde, prior to being acquired on an AriaFusion cell sorter (Becton Dickinson). CD8 T cells attached to peptide-HLA multimers were sorted into tubes saturated with 2% BSA, by gating on PE-positive CD8 populations. In order to reveal the peptide specifies of the sorted T cells, the attached DNA barcodes were amplified using PCR (Taq PCR Master Mix Kit, Qiagen), purified (QIAquick PCR Purification kit, Qiagen), and read by DNA sequencing using Ion Torrent PGM 314 or 316 chip (Life Technologies) at either Sequetech (USA) or GeneDx (USA). To process the barcode sequencing data we used the software package Barracoda (<https://services.healthtech.dtu.dk/service.php?Barracoda-1.8>), which counts barcode reads relative to background, and assigns read counts and statistics to the corresponding peptide specificity.

## Functional evaluation of antigen-specific T cells

To investigate the immunogenic potential of the detected peptides, we cultured the respective patient’s bone marrow cells with scaffolds consisting of peptide-MHC complexes bound to a dextran backbone with attached IL-2 and IL-21, in order to expand the fraction of specific T cells. Half of the cells were cultured using MHCs loaded with peptides detected from the barcode screening experiment, and half with peptides that were predicted binders, but didn’t show up during the screening. The cells were cultured for 10 days in X-Vivo media + 5% human serum. Media was renewed and new peptide-MHC scaffolds were added every 3 days. Following the 10 days of culture, cells were re-stimulated with soluble free peptides at a concentration of 1 µg/ml, after which cells were permeabilized and stained for intracellular cytokines (ICS) in accordance with manufacturer protocols.
